# An automated and robust method for modelling X-ray beamlines with plane grating monochromators

**DOI:** 10.1107/S1600577525003200

**Published:** 2025-05-13

**Authors:** Patrick Yuheng Wang, Murilo Bazan da Silva, Georg Held, Hongchang Wang, Kawal Sawhney, Andrew C. Walters

**Affiliations:** a Diamond Light Source Harwell Science and Innovation Campus Didcot OxfordshireOX11 0DE United Kingdom; b EaSTCHEM School of Chemistry University of Edinburgh EdinburghEH9 3FJ United Kingdom; University College London, United Kingdom

**Keywords:** soft X-rays, ray tracing, monochromators, plane gratings, simulation

## Abstract

The plane grating monochromator (PGM) is an optical instrument used in the majority of soft X-ray beamlines. Despite its ubiquity, the PGM efficiency can easily be overestimated, because the geometry of many modern PGMs can lead to unexpected blocking of the beam. We have developed a new workflow in Python for simulating PGMs, thus extending the capabilities of *SHADOW3*, a well established ray tracing software tool. We have used our method to simulate the flux on branch C of the Versatile Soft X-ray (VerSoX) beamline B07 at Diamond Light Source. The simulation results demonstrate qualitative agreement with the experimental measurements, confirming the robustness of the proposed methodology.

## Abstract 2

A new set of tools to allow more accurate modelling of plane grating monochromators in *SHADOW3* are presented.

## 1. Introduction

A significant proportion of soft X-ray beamlines use a plane grating monochromator (PGM) to provide a monochromatic X-ray beam. PGMs consist of a mirror followed by a plane grating, producing an energy-dispersed X-ray beam with a fixed trajectory (Kunz *et al.*, 1968 ▸; Follath, 2001 ▸; Wang *et al.*, 2025 ▸). Such monochromators combine ease of use with substantial flexibility in operation compared with alternative soft X-ray monochromators.

The design of the PGM directly impacts the performance of the beamline. Beamline properties such as the energy resolution and the photon flux are critical to the success of experiments such as X-ray absorption, X-ray emission or X-ray photoemission spectroscopies (XAS, XES, XPS) and are routinely predicted using ray tracing simulations. PGMs work by using the dispersive property of the grating, which is described by the grating equation,
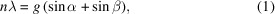
where *n* is the order of diffraction, λ is the wavelength of the outgoing radiation, *g* is the grating period, and α and β are the angles of incidence and diffraction, respectively. For any given values of *n*, λ and *g* there are multiple pairs of (α, β) values that can satisfy equation (1). One way to specify a unique solution is to define a property of the PGM known as the fixed-focus constant, *c*_ff_ (Petersen, 1982 ▸),
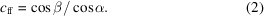
We can then use *c*_ff_ and λ (or photon energy) to define unique values of α and β for a grating with a given period *g* operating in the *n*th order.

It is possible to simultaneously diffract multiple diffraction orders from a grating, which can lead to higher-order contamination issues. This is a problem frequently encountered in the soft X-ray range (Waki *et al.*, 1989 ▸; Frommherz *et al.*, 2010 ▸; Sokolov *et al.*, 2018 ▸). Such issues are ideally addressed during the design of the beamline, which can then be mitigated by optimizing the grating parameters and/or the PGM mirror coatings.

Currently, there are several challenges surrounding the modelling of PGMs. Most PGMs that allow the user to vary the PGM mirror grazing angle use a mechanical scheme first used in SX-700 PGMs (Riemer & Torge, 1983 ▸). While this design is very flexible, its geometry is rather complex and unintuitive (Wang *et al.*, 2025 ▸), and as such blocking can occur during operation, making these SX-700 type PGMs inherently difficult to model in ray tracing software. Second, incomplete modelling can mean that issues such as higher-order contamination are not fully appreciated during the design phase, which can lead to challenges in operation. Third, since collimated PGM schemes allow both the energy and *c*_ff_ to be selected independently, there is a large 2D parameter space available that would be rather time-consuming to explore using traditional approaches.

At Diamond Light Source, the ray tracing software of choice is *SHADOW3* (Sanchez del Rio *et al.*, 2011 ▸), which is used by a significant fraction of the synchrotron optics community. Although other software packages are available, such as *RAY* (Schäfers, 2008 ▸) and *xrt* (Klementiev & Chernikov, 2014 ▸), we have concentrated on the implementation of our methodology in *SHADOW3*.

By design, *SHADOW3* has no *a priori* knowledge of the global optical setup and, therefore, is restricted by the assumption of sequentiality. Due to this design principle of the underlying codebase, ray tracing is done in the sequence specified by the user. In the operation of a typical SX-700 type PGM, the correct sequence met by the rays is the mirror followed by the grating. In scenarios where the rays are blocked by the upstream corner of the grating before the mirror or by the downstream corner of the mirror after the grating (Wang *et al.*, 2025 ▸), the sequence is broken. *SHADOW3* is unaware of the blockages and will transmit 100% of the rays. This is problematic when simulations are performed for larger energy ranges; *SHADOW3* will report an overestimated intensity in certain configurations of SX-700 type PGMs. Note that for brevity we refer to SX-700 type PGMs as simply PGMs in the remainder of this work.

A different, but nevertheless important, limitation is the location and the size of the beam as it impinges on the mirror and grating. The typical geometry of a PGM means that the location of the beam footprint on the mirror changes as a function of the incident angle θ. As real mirrors have finite length, this means that, if θ is not within a certain range, part (or all) of the beam will not be transmitted by the PGM. Moreover, in certain energy/*c*_ff_ combinations, the footprint made by the beam on the optical surface of the grating may become larger than that of the grating itself, leading to a loss of flux. Compared with self-blockages, these two scenarios are much more easily handled within *SHADOW3*, as they do not break the assumption of sequentiality described earlier.

In this article, we build on our previous work (Wang *et al.*, 2025 ▸), in which analytical expressions of various geometrical quantities of a PGM were derived. With some modifications and extensions presented here, these expressions can be used in *SHADOW3* to fully model the nuances of the PGM geometry for transmission calculations and more. We also present a programming pipeline and the corresponding code which could be used to simulate any PGM-based beamline in the future. The solution we propose is fully autonomous in modelling all aspects of the PGM and, as such, can fully automate scans over energy, *c*_ff_, order of diffraction, *etc.* This is in direct contrast to how simulations like these are often carried out at present, where manual input is typically needed at multiple stages in the process. The validity of this methodology has been demonstrated via direct comparison of simulation results with flux measurements performed at the B07c beamline (Held *et al.*, 2020 ▸) at Diamond Light Source.

## 2. Methodology

**Figure 1:**
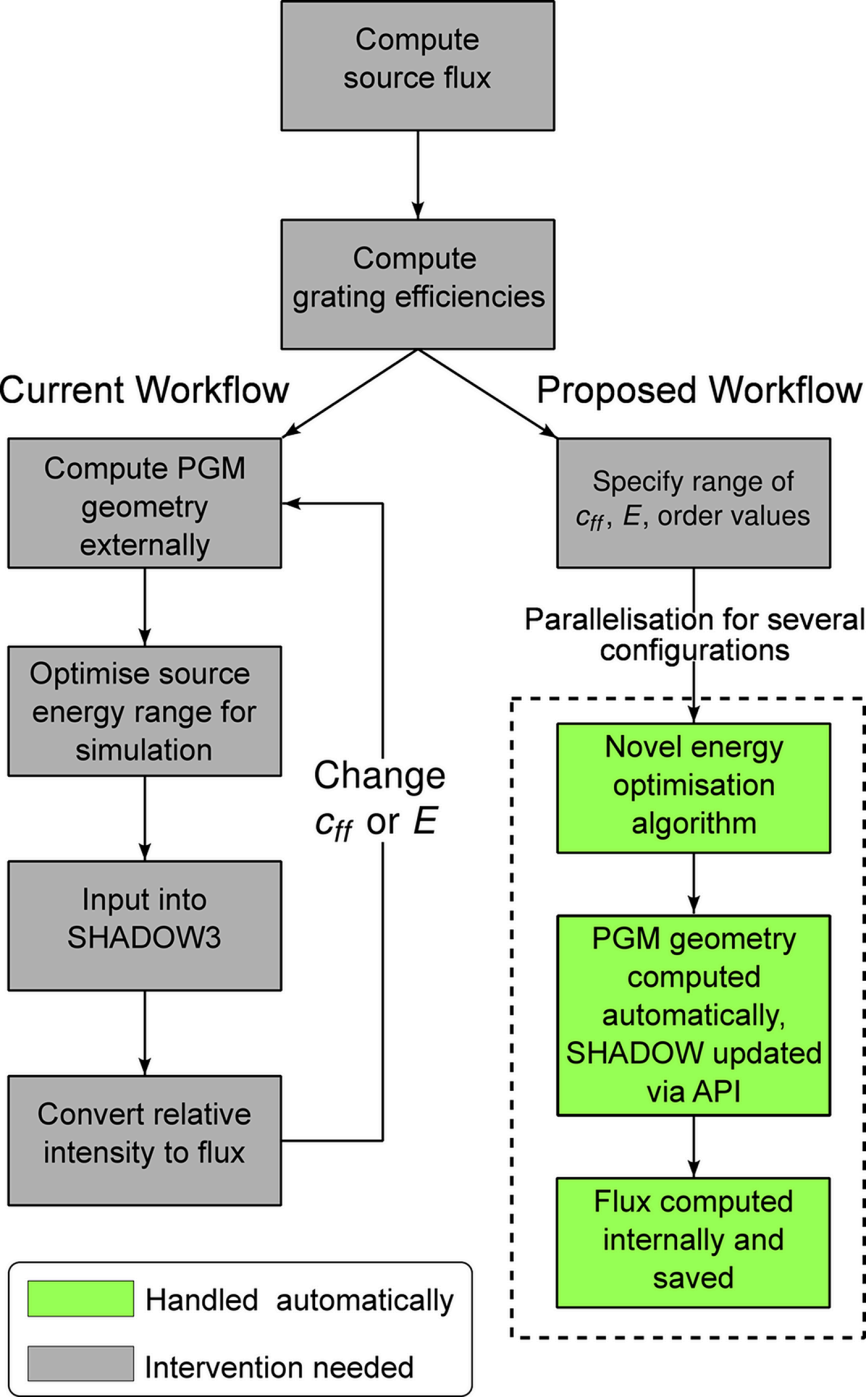
The current standard workflow for carrying out PGM transmitted flux simulations as well as the proposed workflow for an integrated model.

A typical current workflow for simulating a beamline with a PGM is presented in Fig. 1 ▸, along with the workflow we propose in this article. In this section, a step-by-step account of the proposed methodology will be given.

### 2.1. Computation of the source flux

At present, it is not possible to use *SHADOW3* to perform flux simulations for bending magnet sources. After ray tracing, the value returned by *SHADOW3* is the intensity, which is the Pythagorean sum of the magnitudes of the electric and magnetic fields of all of the rays that have been simulated. This intensity can then be scaled to flux in units of photons per second by using externally computed data. We have used *SPECTRA* (Tanaka, 2021 ▸) to calculate the bending magnet source flux for the simulations for B07c. Fluxes are obtained by scaling the ray traced intensity to the flux computed by *SPECTRA* while correcting for the energy bandwidth.

### 2.2. Computation of mirror and grating efficiencies

After propagation – either via reflection or diffraction – from each optical element (OE), some rays may be lost and the overall intensity will therefore be reduced. For mirrors, reflectivities depend on the electronic properties of the mirror coating as well as the roughness of the mirror surface. These can be computed natively in *SHADOW3* using the preprocessors *PREREFL* (Lai & Cerrina, 1986 ▸) and *WAVINESS* (Sanchez del Rio & Marcelli, 1992 ▸), respectively.

Currently, it is not possible to perform grating efficiency calculations in *SHADOW3*. In the calculations performed for B07c, the grating efficiencies of the 400 and 600 lines mm^−1^ gratings have been computed using *MLgrating* (Walters *et al.*, 2024 ▸). The grating efficiency is a function of energy, order and *c*_ff_; it is therefore required that the grating efficiency for each combination of energy and *c*_ff_ be computed separately. The geometrical properties of the gratings, as measured by the manufacturers, are presented in the supporting information. The grating efficiencies at different values of energy and *c*_ff_ were tabulated into JSON files, which can be stored and read easily. The grating efficiencies were calculated for more than 60000 combinations of energy, order and *c*_ff_ for each grating. These calculations took approximately 6 h per grating on a laptop with a six-core Intel Core i7-10810U @ 1.10 GHz.

### 2.3. Optimizing the simulated energy range

To minimize the number of lost rays in the ray tracing simulation, the energy range over which the X-ray source is simulated needs to be optimized. A typical rule-of-thumb is that the energy range of the source should be at least double the energy resolution [full width at half-maximum (FWHM)] achieved by the PGM combined with the exit slit. In this way, one ensures that the energy range of the source is not significantly limiting the simulated energy resolution of the beamline, while ensuring that one does not waste computing resources on many rays that will not be transmitted through the exit slit.

Here we present an automated approach to find an appropriate energy range for the source for any beamline configuration (*E*, *c*_ff_, *n* and exit slit opening). An optimal energy range can be found by a simple while-loop. Starting with an initial energy range for the source, ray tracing with a reduced number of rays is carried out. The energy FWHM of the final beam is doubled and used as the energy range for the next iteration. We then iterate until the energy resolution converges to within a certain predefined tolerance relative to the previous iteration. This method is sufficiently flexible and fast that it can be done on-the-fly during real simulation runs. A scatter plot of an iteration process is included in the supporting information.

### 2.4. Geometry of the PGM and input to *SHADOW3*

To be able to correct for geometrical blockages of the PGM and accurately simulate it in *SHADOW3*, it is important to correctly inform *SHADOW3* of the coordinates of the optical elements (OEs) that define the PGM (a plane mirror and a plane grating) within the reference frames of *SHADOW3*.

There are two primary areas of concern: (1) when the incident angle becomes too small or the beam height becomes too large, the entire beam footprint will not fit the optical surface of the mirror or grating, leading to a loss of flux, and (2) where there are geometrical blockages, caused by the mirror or the grating, the beam will be partially or completely blocked.

In *SHADOW3* the distances and angles of the OEs are defined relative to the OEs that precede them, and the first OE is defined relative to the source. One can define the size of the optical surface directly in *SHADOW3*. In the context of a PGM, this information would simply be the sizes of the optical surfaces of the mirror and grating, which are known. This input can be easily done by setting the attributes for the appropriate OE. Throughout the following, we have inputted the real physical sizes of the optics into *SHADOW3* and have not attempted to make any distinction between these dimensions and the dimensions of their clear apertures.

### 2.5. *SHADOW3* OE offsets

**Figure 2:**
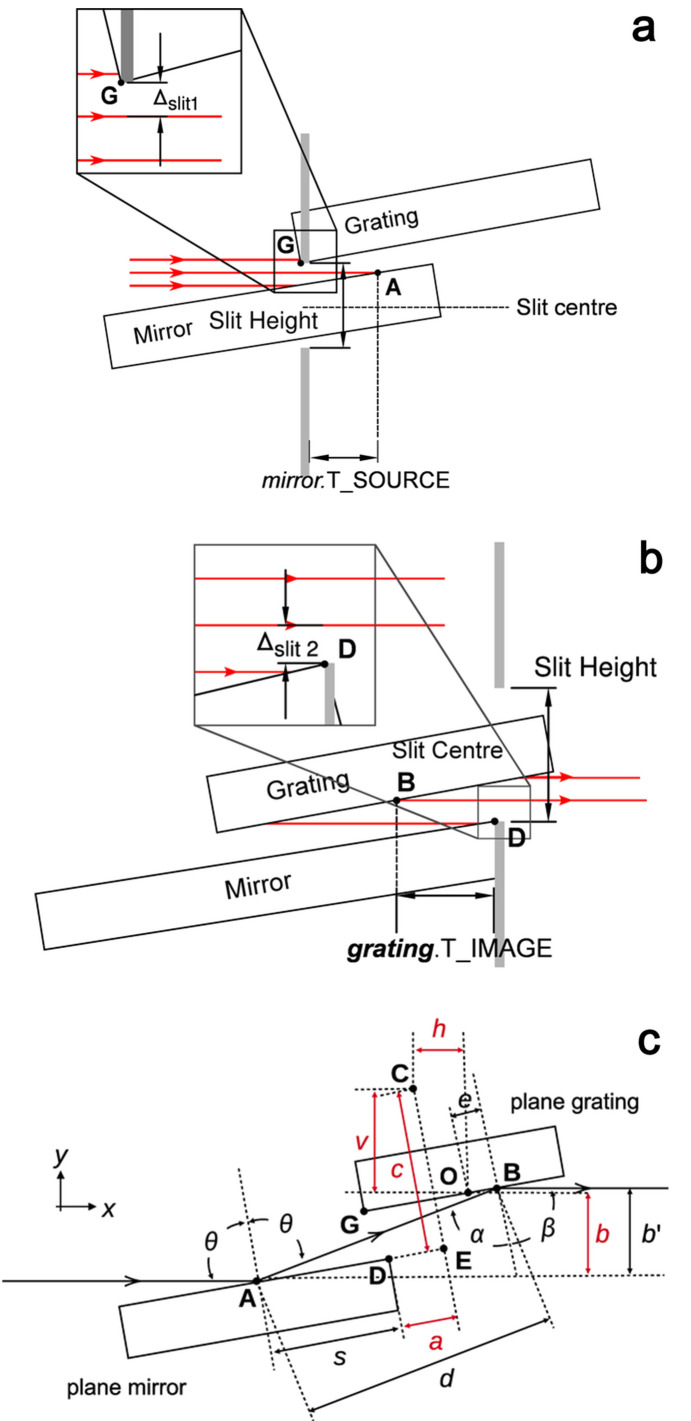
(*a*) Illustration of the slit positioning in *SHADOW3* for blocking by the grating. (*b*) Illustration of the positioning of the second fictitious slit to model beam blockage by the mirror. (*c*) A schematic of the PGM geometry which contains geometrical quantities used in the derivation [reproduced from Wang *et al.* (2025 ▸)].

The position of any OE in *SHADOW3* is defined relative to the centre of the beam. In a typical PGM, the centre of the beam is placed as close to the centre of the grating as possible by design, but the centre of the beam on the mirror changes position as a function of grazing angle. By defining the correct size of the mirror OE in *SHADOW3*, along with the correct mirror offset relative to the beam, scenarios where the beam falls off the mirror can be accounted for in *SHADOW3*. This offset is set through the 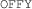 attribute of the mirror. Using the parameters presented in Fig. 2 ▸(*c*) (Wang *et al.*, 2025 ▸), it can be shown that the 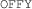 parameter is given by
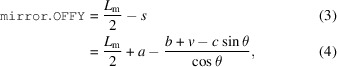
where *L*_m_ is the length of the mirror. The parameters *a*, *b*, *c* and *v* are known offsets of the PGM, and θ = (α − β)/2.

Similarly, for the grating, the required offset 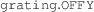 can be shown to be
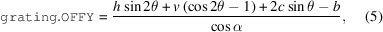
where all quantities are known. The correct input of these offsets, along with the correct dimensions of the optics, allows *SHADOW3* to handle cases where the beam footprint is partially off the mirror or the grating.

However, enabling *SHADOW3* to handle blockages is not trivial. Formally, OEs in *SHADOW3* are treated as infinitely thin planes with finite sizes. Without modifying the underlying codebase of *SHADOW3*, we have implemented the following workaround. Two additional fictitious slits are included in the simulated PGM, where one of the defining blades of the first slit is placed at the upstream corner of the grating, while one of the defining blades of the second slit is placed at the downstream corner of the mirror. To correctly define these two points, the distances from these fictitious slits to the next optical element 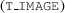 also need to be known. This quantity is essential in correctly accounting for the beam size when a non-collimated beam passes through the PGM. In practice, the two parameters that need to be specified for the slits are the vertical offset from the centre of the slit with respect to the centre of the beam and the horizontal distance from the slit to the next OE.

### 2.6. Blockage by the grating

A slit is defined in *SHADOW3* with a height and location of the centre of the opening relative to the centre of the beam. To account for blockage by the grating, a slit is introduced in the optical setup as shown in Fig. 2 ▸, where we have defined the point **G** to be the bottom left corner of the grating. Programmatically, the slit height is an arbitrary choice, as long as it is set larger than the height of any possible synchrotron beam. Here we chose a value of 1000 mm. As illustrated, the slit should be translated down so that the bottom of the upper obstruction is placed at **G**. The quantity of interest is therefore the vertical distance from the centre of the beam to **G**, which is denoted as Δ_slit1_. Defining the origin to be at the centre of the grating optical surface, **G** can be expressed as
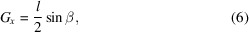

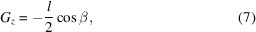
where *l* is the length of the grating. An expression for Δ_slit1_ can then be derived,
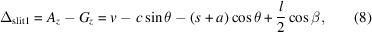
where *A*_*z*_ and *G*_*z*_ are the *z* components of the points **A** and **G**, respectively. The 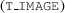 parameter is then the horizontal distance from **G** to **A**,
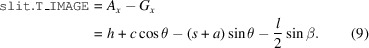


### 2.7. Blockage by the mirror

Similarly, for the blockage by the mirror, the location of the downstream mirror corner (**D**) must be known. This position needs to be defined relative to the centre of the beam **B**; if we once again define the origin to be at the centre of the optical surface of the grating, the *x* and *z* components of **B** can be extracted,
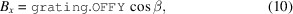

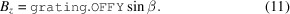
The required offsets are then the horizontal and vertical distances from the two points, 
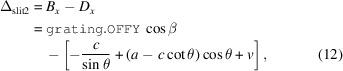

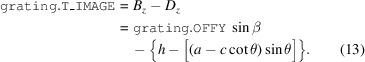
We note that 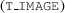 for the second slit is not a property of the slit but a property of the grating OE, as in *SHADOW3* the OEs are defined relative to the previous OE via the attribute 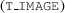. Alternatively, we could have defined this distance relative to the second slit with the attribute 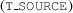. Thus, the ray trace sequence for the PGM is as follows:

(i) Slit 1, to handle blockage by the upstream edge of the grating. Defined by Δ_slit1_ and 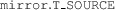 (distance to the mirror, equivalently, 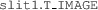).

(ii) Mirror, to deflect the rays to meet the grating. Defined by 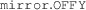 and 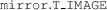 (distance to the grating).

(iii) Grating, to disperse the rays. Defined by 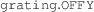 and 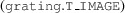 (distance to slit 2).

(iv) Slit 2, to handle the blockage by the downstream corner of the mirror. Defined by Δ_slit2_.

All parameters needed to fully model a PGM using *SHADOW3* are therefore known:

(i) Sizes of the OEs (native to *SHADOW3*).

(ii) Angles α and β [native to *SHADOW3*, but calculated using *pyplanemono* (Wang *et al.*, 2025 ▸)].

(iii) Mirror reflectivities [native to *SHADOW3* (Lai & Cerrina, 1986 ▸)].

(iv) Grating efficiencies [calculated using *MLgrating* (Walters *et al.*, 2024 ▸)].

(v) Mirror and grating translations relative to the beam (this work).

(vi) Positions of the two fictitious slits (this work).

A model of the B07c beamline at Diamond Light Source (Held *et al.*, 2020 ▸) was constructed in *ShadowOui* (Rebuffi & Sanchez del Rio, 2016 ▸) and, using the *Info* post-processor, a Python script was generated. The *pyplanemono* Python library (Wang *et al.*, 2025 ▸) was extended with an interface to the *SHADOW3* Python API. This configures all the OEs appropriately with the correct angles and offsets within the simulation. Scans of energy, *c*_ff_ and diffraction order were performed simultaneously using the Python internal multiprocessing library. By running 25 processes concurrently, the complete simulation using precomputed grating efficiencies took about 12 h to run on a machine with a 48-core Intel Xeon CPU E5-2650 v4 @ 2.20 GHz.

## 3. Results

To understand the extent of higher-order contamination on the B07c beamline, the flux for diffraction orders one through four were simulated for an existing 400 lines mm^−1^ Au-coated laminar grating (Held *et al.*, 2020 ▸). The energy ranges for the different orders were scaled by the order; energies ranged from 300 eV to 3000 eV for the first order (covering most of the core beamline range), up to 6000 eV for the second order, 9000 eV for the third and 12000 eV for the fourth. Simulations were performed at these energies over a range of *c*_ff_ values between 1.05 and 3.0.

It is common for PGMs to have several mirror coatings and several gratings that can be exchanged during operation. The PGM at B07c has several possible mirror and grating combinations. For the sake of conciseness, here we only present results for the 400 lines mm^−1^ laminar grating and Pt mirror combination; however, the 600 lines mm^−1^ blazed grating and Pt mirror combination were also fully modelled, and the results are included in the supporting information. We note that all the non-PGM mirrors on the B07c beamline have Rh coatings. The maximum energy of B07c is defined by the fixed grazing angle (1.1°) of the first mirror M1c, which is located before the PGM.

**Figure 3:**
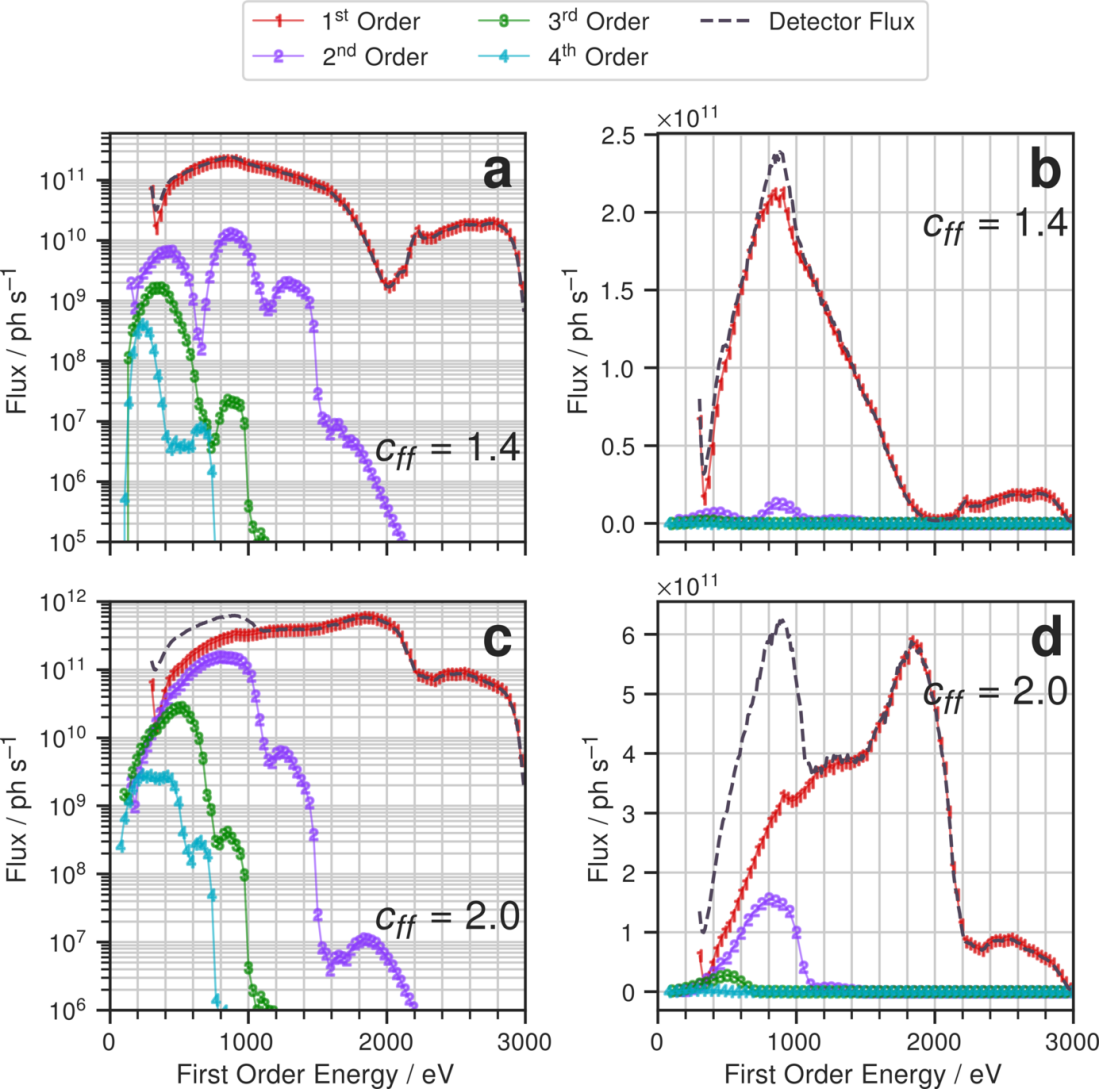
Flux simulations carried out for orders 1–4 at (*a*) *c*_ff_ = 1.4 and (*c*) *c*_ff_ = 2.0. Both are for the B07c Pt mirror and 400 lines mm^−1^ grating combination. Note that the *x*-axis plots first order energy, *i.e.* real photon energy divided by order. Panels (*b*) and (*d*) show the same data but on a linear *y*-scale.

Fig. 3 ▸ plots the simulated flux at the sample at *c*_ff_ = 1.4 and 2.0, which are two values routinely used on the beamline. Note that the *x*-axis plots the first order energy. Due to the nature of diffraction from a grating, a PGM in a certain geometry will also transmit integer multiples of the first order energy for higher orders. As such, in order to understand the total flux transmitted at a given first order energy setting, we must scale the energy axes of the higher order flux curves by the reciprocal of their respective orders; the scaled energies are referred to as *first order energy* in subsequent discussions and figures.

Comparison of our simulation with measured flux also requires the introduction of a *detector flux*. The measured signal of photodiodes, which are typically used to measure photon flux at X-ray beamlines, is proportional to the product of flux and photon energy, *i.e.*
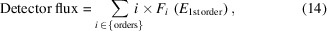
where 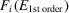 is the flux of the *i*th order at first order energy. The detector flux gives a useful metric, as it much more closely resembles what the beamline can measure. This quantity is plotted in Fig. 3 ▸ with dashed lines.

Figs. 3 ▸(*a*) and 3(*b*) show that the lower value *c*_ff_ of 1.4 contains little higher-order contamination without overly sacrificing first order flux below 1000 eV. The equivalent plots are presented for *c*_ff_ = 2.0 in Figs. 3 ▸(*c*) and 3(*d*). The detector flux is significantly higher, almost double that at *c*_ff_ = 1.4, but the contribution from higher orders accounts for at least 50% of the detector flux below 900 eV and up to 80% of the detector flux at the very lowest energies. We note that, without the results of our integrated modelling, it would be very difficult to methodically choose an optimal *c*_ff_ value because most of the methods of flux measurements are not energy-resolved.

Furthermore, in Fig. 3 ▸, it is observed that with increasing order the flux decreases. Although the spectrum of the bending magnet produces higher flux at higher energy (up to 4000 eV for B07c), the grating efficiency rapidly reduces with increasing diffraction order. This means that, despite the advantage higher orders have from the bending magnet source, they are suppressed by the grating significantly. However, second order flux still contributes appreciably to the total flux at the higher *c*_ff_ of 2.0 [see both Figs. 3 ▸(*c*) and 3 ▸(*d*)].

**Figure 4:**
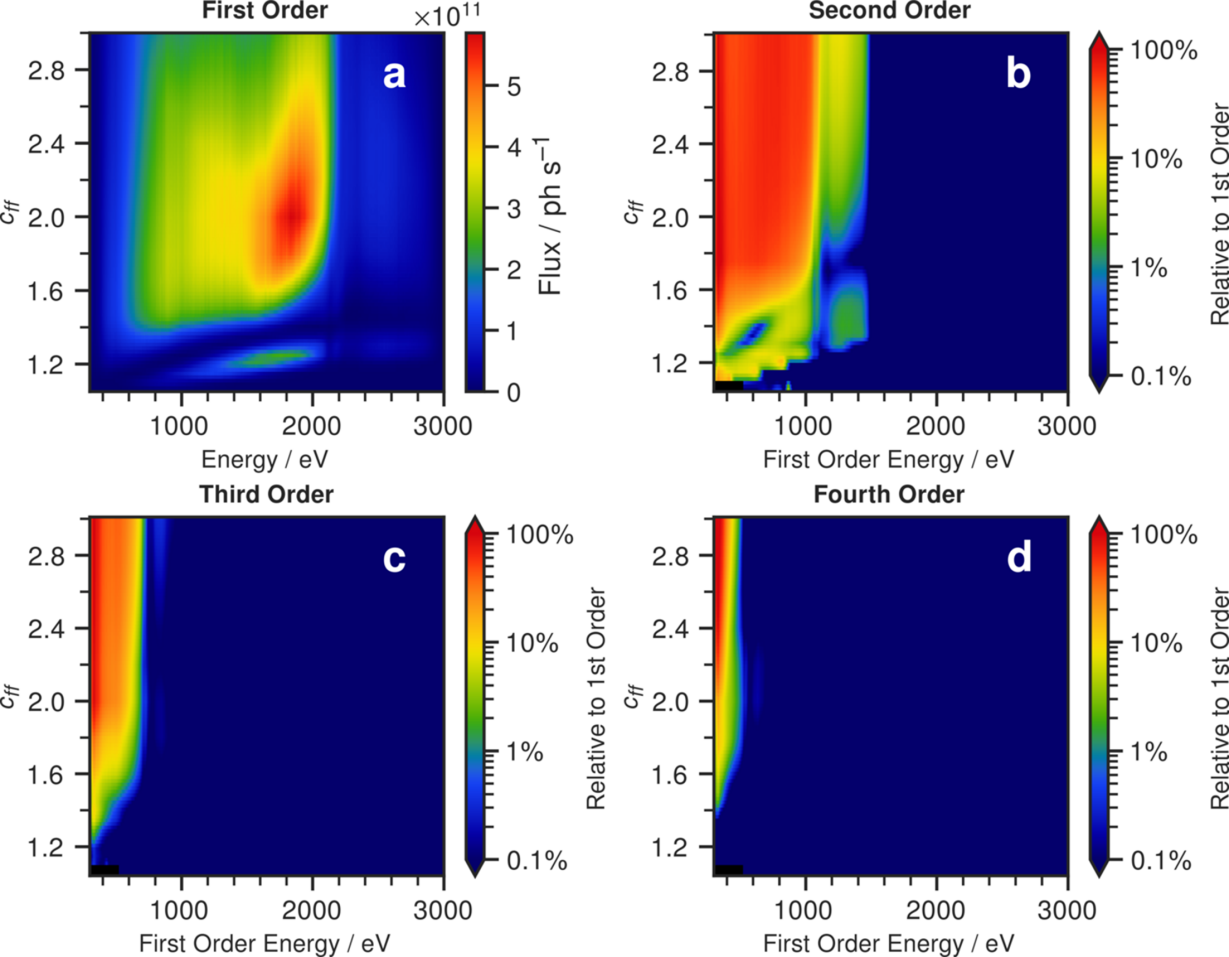
Results presented as four heat maps of (*a*) first order flux as a function of *c*_ff_ and energy; and second (*b*), third (*c*) and fourth (*d*) order flux relative to the first order flux as a function of *c*_ff_ and first order energy. Note the logarithmically scaled colour bars.

The extent of the higher order transmission can be visualized in the 2D parameter space of energy and *c*_ff_. In Fig. 4 ▸ we have followed the presentation style used in a previous study (Sokolov *et al.*, 2016 ▸), where the ratios of the transmitted fluxes of orders two, three and four relative to the first order flux are plotted as a function of energy and *c*_ff_. From Fig. 4 ▸, one observes that the second order contributes significantly more than the third and fourth orders. Up to a first order energy of ∼1500 eV (corresponding to the Rh *L*-edge in second order), suppression of the second order is especially poor, with contamination above 25%. This is problematic, as this is in the core energy range of the beamline. The beamline has found empirically that operating at a *c*_ff_ of 1.4 reduces the higher-order contamination by a substantial amount. Our simulations validate that decision, as Fig. 4 ▸(*a*) shows that a *c*_ff_ value of 1.4 resides just at the lower edge of the region of high first order flux [Fig. 4 ▸(*a*)] while minimizing the second order flux [Fig. 4 ▸(*b*)].

**Figure 5:**
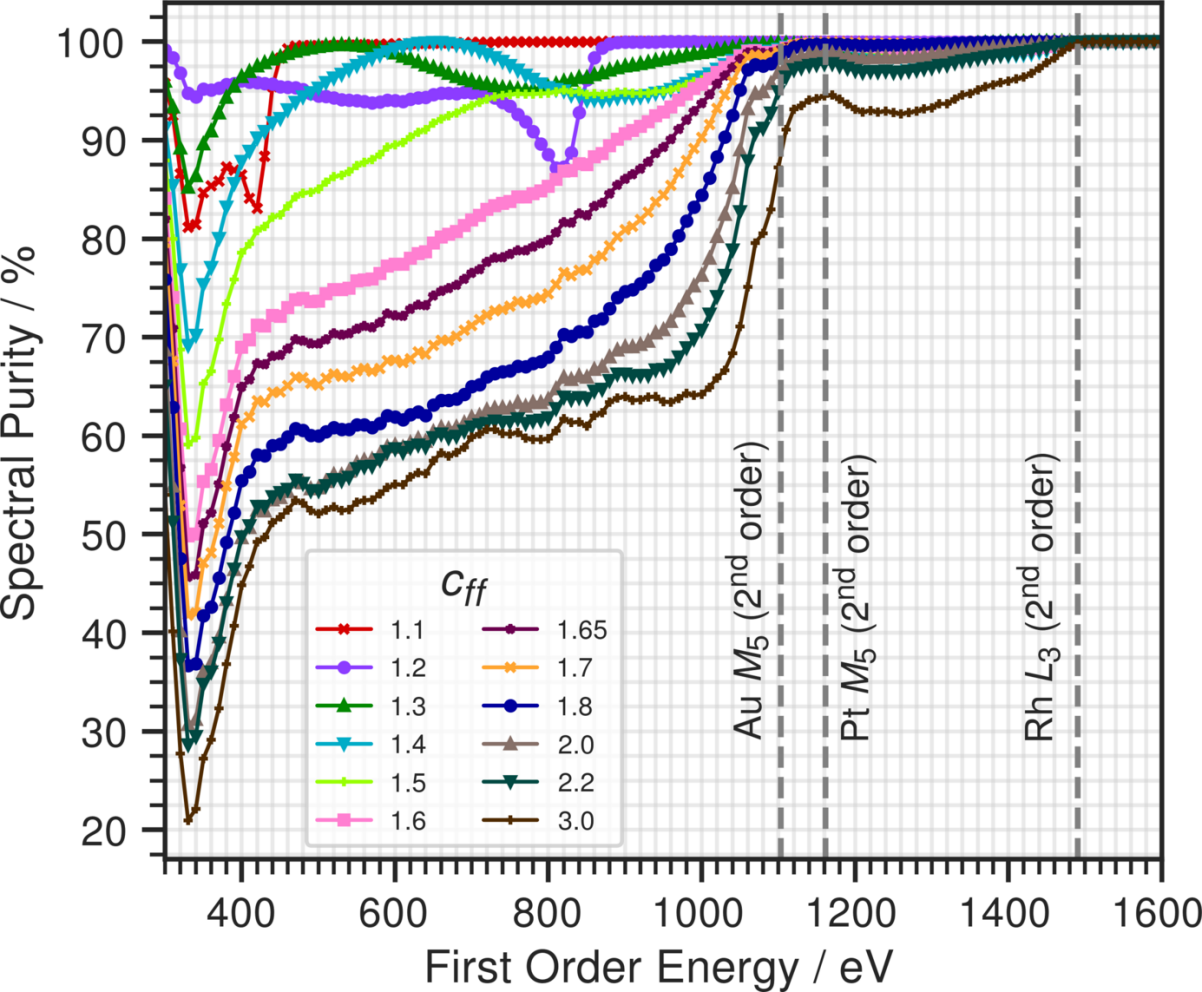
Spectral purities of the beam after the final OE as a function of energy. The grey dotted lines at 1100 and 1500 eV in first order energy correspond to absorption edges in the second order at 2200 eV and 3000 eV.

Fig. 4 ▸ also shows a general trend of increased contamination with increasing *c*_ff_. To compare the performance between different values of *c*_ff_, we use an established definition of spectral purity (Sokolov *et al.*, 2016 ▸),
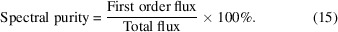
The contribution of first order flux should therefore ideally be as close to 100% of the total flux. A selection of spectral purities at different values of *c*_ff_ is plotted in Fig. 5 ▸. The minimum in the spectral purity observed at all values of *c*_ff_ at ∼320 eV is due to the first order flux being suppressed by the Rh *M*_5_ edge. The deterioration of the spectral purity from *c*_ff_ = 1.1 to *c*_ff_ = 1.8 at lower energies is much more rapid compared with higher values of *c*_ff_, where the spectral purity slowly plateaus at around 60% up to 1100 eV. The stagnation of the decrease in spectral purity can be explained by the fact that the angles are changing much more drastically as a function of *c*_ff_ at lower values of *c*_ff_, significantly changing the reflectivity of the PGM mirror.

Drastic improvements in spectral purity are observed independently of *c*_ff_ above 1100 eV and again above 1500 eV. The first improvement can be accounted for by the *M*_5_ edges of Pt and Au at around 2200 eV (1100 eV in first order energy) due to the mirror and grating coatings, respectively, greatly suppressing the transmission of second order flux. Similarly, one observes an improvement at 1500 eV first order energy with Rh *L*_3_ absorption. The mirrors and grating are acting as *de facto* low pass filters, transmitting lower energies while suppressing higher energies. The low pass filter property of mirror reflectivities has been previously exploited to build higher order suppressors (Sokolov *et al.*, 2018 ▸; Frommherz *et al.*, 2010 ▸). Figs. 3 ▸(*c*) and 3 ▸(*d*) show that most of the contamination at *c*_ff_ = 2.0 comes from the second order when the first order energy is between 500 and 1000 eV. There is a smaller contribution from the third order when the first order energy is between 300 and 600 eV.

**Figure 6:**
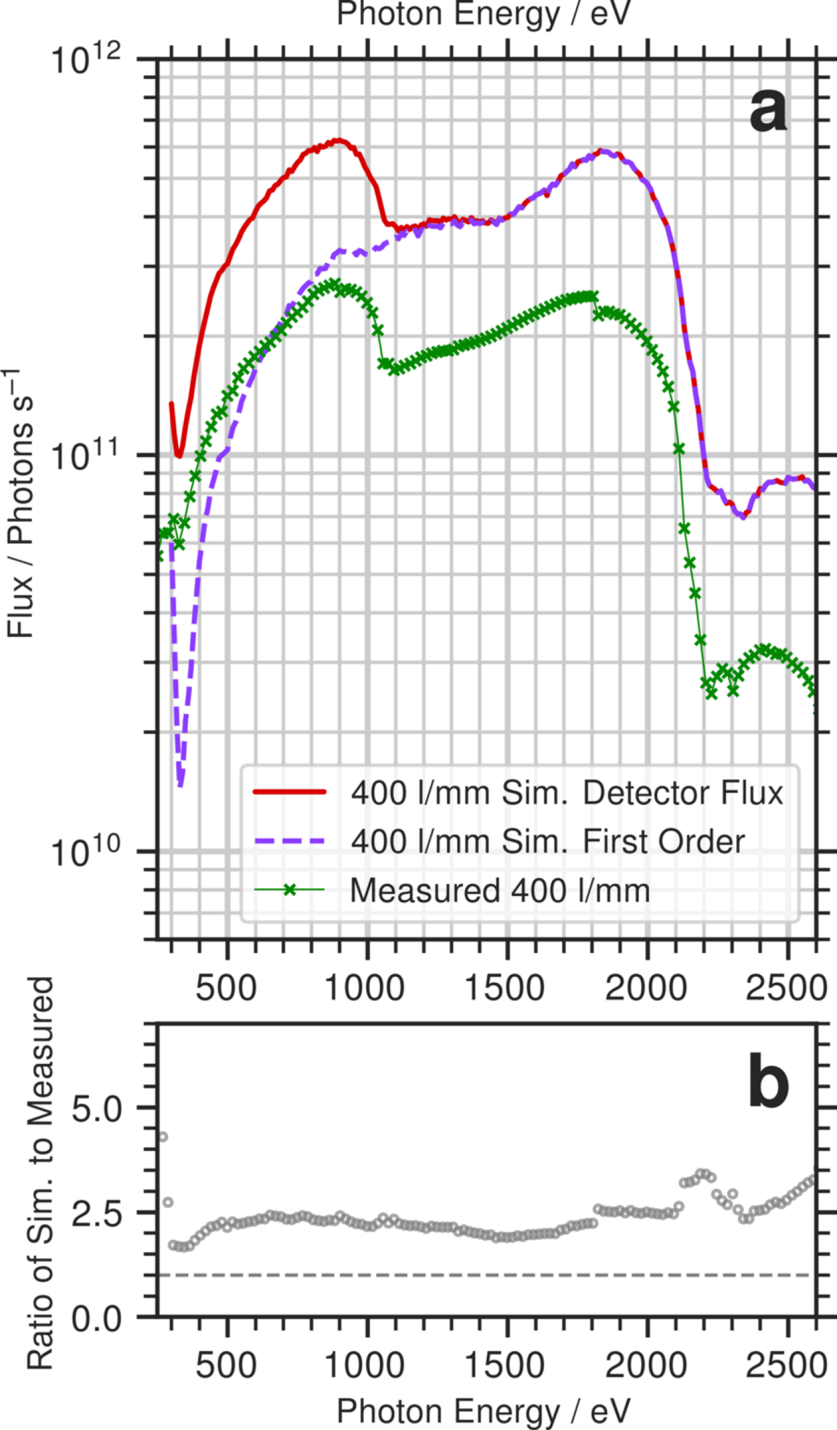
(*a*) Simulated detector flux (red) and the first order component (dashed purple) compared with the measured flux at a *c*_ff_ value of 2 (Held *et al.*, 2020 ▸). (*b*) Ratio of the simulated detector flux to the measured flux in (*a*).

Held *et al.* (2020 ▸) report the detector flux for B07c at the sample position at a *c*_ff_ of 2. The simulated detector flux, the simulated first order flux and the measurements reported by Held *et al.* (2020 ▸) are presented in Fig. 6 ▸(*a*). Fig. 6 ▸(*b*) plots the ratio of the simulated flux at the sample position to the measured flux. We note that for this particular case our modelling of the PGM geometry (described in Section 2.5) indicates that only part of the beam is reflected from the PGM mirror above 1900 eV, highlighting the importance of accurately representing the PGM in our model.

In Fig. 6 ▸(*a*), the simulated detector flux very closely resembles that of the measurement. The drops in the measured flux around 920 eV and 1840 eV are artefacts of the silicon photodiode used to measure the flux and are due to the Si *K*-edge viewed in second and first order, respectively. Compared with the first order flux shown in Fig. 6 ▸(*a*) (dashed purple line), the measured flux has discernible features that negate the possibility of a pure first order transmittance.

However, the absolute flux in units of photons per second is different between calculation and measurement. While the measured flux peaks at 2 × 10^11^ photons s^−1^, the simulation peaks at around 6 × 10^11^. We believe that the most prominent factor in this difference is related to bake-out induced distortions in the horizontally deflecting first mirror M1 which have been partially corrected by modifying the temperature of the cooling water (Hand *et al.*, 2019 ▸). The remaining aberrations in the beam are likely to result in a large, non-Gaussian horizontal beam profile at the exit slit. As the horizontal opening of the exit slit was set at 0.8 mm (Held *et al.*, 2020 ▸), a significant part of the beam may not have been transmitted through the exit slit. Note that the simulations presented here have assumed that the M1 slope errors are those achieved by the supplier when the mirror was originally manufactured. It is also not particularly surprising that there are larger differences around the Pt and Au absorption edges (above 2100 eV), as here subtle differences in the surface chemistry can make a big impact on the X-ray reflectivity.

Experimentally, higher order contamination can be detrimental to the quality of the data. Indeed, the beamline now operates almost exclusively at a *c*_ff_ of 1.4 in the energy range below 1200 eV (*cf*. Fig. 3 ▸). Using the detailed results from the integrated model presented here, an optimized mode of operation can be proposed.

**Figure 7:**
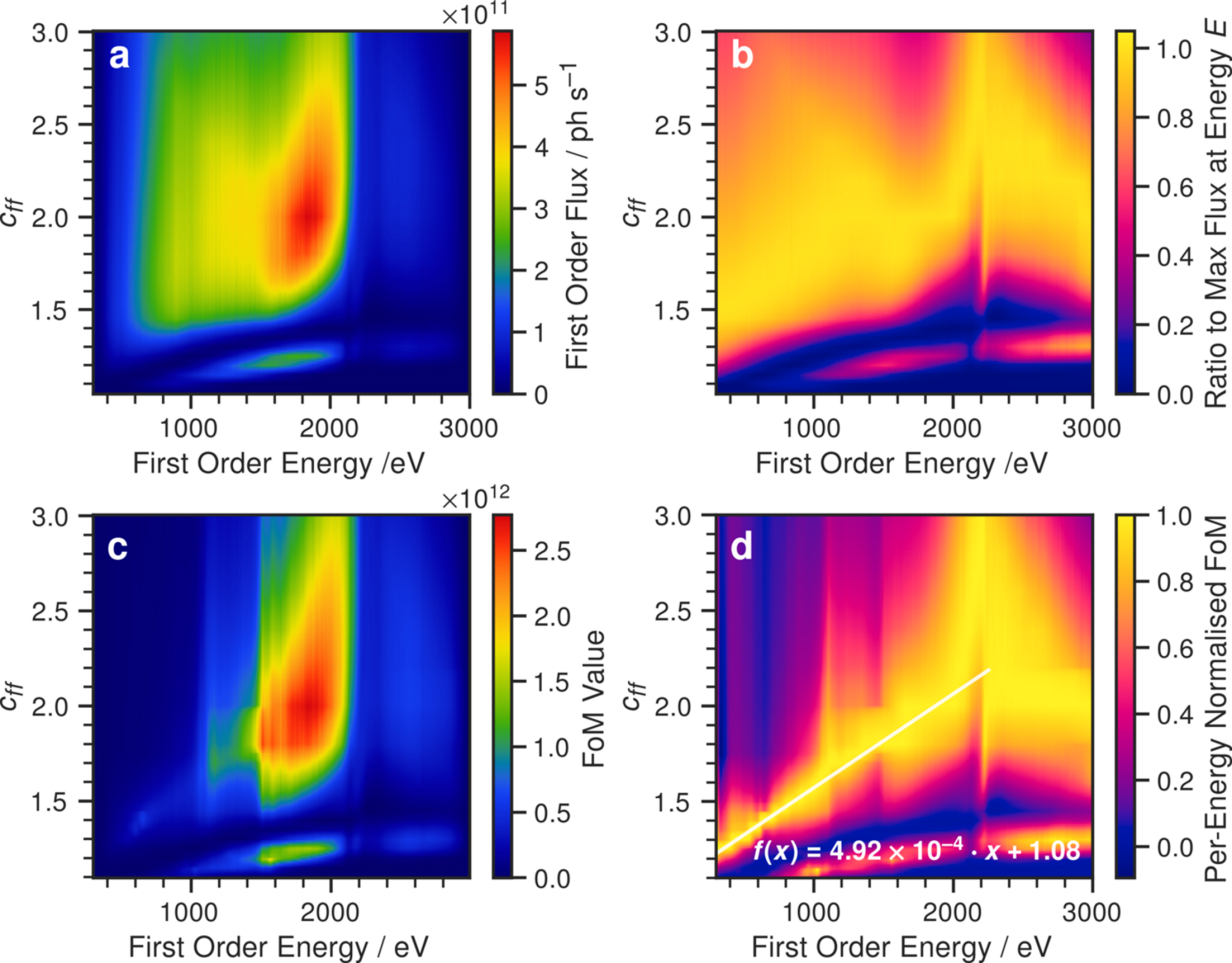
(*a*) First order flux as a function of energy and *c*_ff_. (*b*) First order flux normalized to per energy. (*c*) The FoM function. (*d*) Data after being normalized at each energy, in the same way as in (*b*). Note the discontinuity at 1100 eV due to the second order Pt and Au *M*-edge absorption; the same absorption occurs at ∼2200 eV for the first order. The semi-transparent line in (*d*) shows a fit through the maxima to offer an accessible ‘rule-of-thumb’.

There are two factors for consideration, one of first order flux and the other of higher-order transmission. As the first order flux is the one desired by the user, it stands to reason that it should be maximized. The first order flux is plotted in Fig. 7 ▸(*a*) as a function of *c*_ff_ and energy. In Fig. 7 ▸(*b*), the same information is shown, but each point was normalized to the maximum flux at the same energy. This allows for the visualization of flux information in the region where the flux is generally low, above the Pt and Au edges. Brighter areas (closer to the value of 1.0) represent energy–*c*_ff_ combinations where first order flux dominates, darker regions the opposite. Generally, from Fig. 7 ▸(*b*), the optimal *c*_ff_ oscillates about a value of ∼2.0. One may therefore be tempted (without knowledge of the extent of higher-order transmission) to operate around that *c*_ff_.

One way to attempt to simultaneously maximize first order flux and maximize higher order suppression is to introduce a figure of merit (FoM) function which combines the two properties. In the design of a higher order suppressor at BESSY-II (Sokolov *et al.*, 2018 ▸; Sokolov *et al.*, 2016 ▸), the following FoM function was proposed,
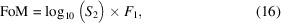

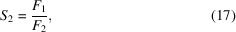
where *F*_1_ and *F*_2_ are the first and second order fluxes, respectively, and *S*_2_ is the suppression of second order. This is a purely empirical equation that attempts to balance the preference between first order flux and higher-order suppression. The choice was made by Sokolov *et al.* to omit orders higher than two, as the grating efficiency decays significantly leading to low transmission and reflectivity, which is in agreement with what we have observed in this work. The FoM presented above has been calculated using our simulation results and is presented in Figs. 7 ▸(*c*) and 7 ▸(*d*).

In Fig. 7 ▸(*c*) the FoM is plotted as written in equation (16), and in Fig. 7 ▸(*d*) the per-energy-normalized equivalent of *c* is shown. Up to around 1500 eV, where first order flux is relatively high [Fig. 7 ▸(*a*)], the transmission of second order is also high and therefore reduces the FoM [Fig. 7 ▸(*c*)]. In Fig. 7 ▸(*d*) a fit is provided with the primary intention of providing a quick, on-the-fly functional form that can be used to estimate the best combination of energy and *c*_ff_ up to 2250 eV. A simple linear function was used, with a best fit slope of 4.92 × 10^−4^ eV^−1^ and an intercept of 1.08. It should be noted that the FoM does not consider the energy resolution, which generally decreases with decreasing *c*_ff_. At lower energies (<1000 eV), the energy resolution provided by the B07c PGM is still relatively high, so operating at low values of *c*_ff_ down to around 1.4 can still provide a sufficiently high energy resolution. However, depending on the user requirements for a given experiment, in some cases it may be beneficial to deviate from the linear fit and increase the *c*_ff_ somewhat to improve the energy resolution.

## 4. Conclusion and outlook

In this article, we have presented a set of analytical expressions and corresponding Python code which extend the capabilities of *SHADOW3* in accurately modelling PGM beamlines. This work provides a collection of tools which optimize the workflow for future simulations of soft X-ray beamlines. The code and documentation are publicly available at https://github.com/MBZN/pyplanemono/.

The newly proposed pipeline was used to carry out a systematic simulation of the B07c beamline at Diamond Light Source. The results presented here are highly convincing in replicating larger structures of the measured energy spectrum of the beamline. Looking at the data holistically, a set of recommendations was made using a figure of merit function described in the literature (Sokolov *et al.*, 2018 ▸). A linear fitting of the maxima of the figure of merit provides a trajectory in energy–*c*_ff_ parameter space that one should follow to maximize first order flux while minimizing higher order transmission.

The modelling presented in this work primarily concentrated on the issue of higher harmonic contamination. However, the established methodology can be applied to simulate other beamline properties of interest, such as the energy resolution. In the simulations presented here, the exit slits of the PGM were fixed to be 100 µm in all cases. In reality, the exit slit opening is adjusted in operation to modify the energy resolution and the flux simultaneously. The software tools described in this work straightforwardly allow comprehensive simulations to be performed as a function of exit slit opening. More broadly, our work allows for extensive automated simulations to be performed which could potentially help to optimize grating designs. The established workflow reduces the amount of human input needed to a minimum, making iterative simulations across energies, *c*_ff_ values, grating line densities *etc.* both time-efficient and also more robust.

## 5. Related literature

The following references, not cited in the main body of the paper, have been cited in the supporting information: Haynes & Lide (2015 ▸); Henke *et al.* (1993 ▸); Peatman (1997 ▸).

## Supplementary Material

Plots of transfer functions as referred to in the manuscript as well as simulations parameters not otherwise mentioned in the main manuscript. DOI: 10.1107/S1600577525003200/ing5010sup1.pdf
