# Supplementary material for: An automated and robust method for modelling X-ray beamlines with plane grating monochromators
Source: J Synchrotron Radiat. 2025 May 13;32(Pt 4):899–907. doi: 10.1107/S1600577525003200 (PMC12236243; doi:10.1107/S1600577525003200)
Supplement: Supplementary file 1 [file s-32-00899-sup1.pdf]

An Automated and Robust Method for Modelling X-ray Beamlines with Plane Grating Monochromators
Supplementary Information

Patrick Yuheng Wang *[1,2], Murilo Bazan da Silva[1], Georg Held[1], Hongchang Wang[1], Kawal Sawhney[1], and Andrew Walters[1]

[1]Diamond Light Source, Harwell Science and Innovation Campus, Didcot, OX11 0DE, U.K.
[2]EaSTCHEM School of Chemistry, University of Edinburgh, EH9 3FJ, U.K.
`patrick.wang@chem.ox.ac.uk`, `andrew.walters@diamond.ac.uk`,
`murilo.bazan-da-silva@diamond.ac.uk`

April 2025

*Now at the Department of Chemistry, University of Oxford

Abstract

In this supplementary information, we describe the visualisation of the transfer function and its applicability in performing soft X-ray ray-tracing simulations. The results for the 600 l/mm grating simulations are presented. We demonstrate that the methodology proposed in our article is capable of modelling the nuances of the plane grating monochromator (PGM) geometry. We also highlight a potential weakness in the package *XRAYLIB* in the soft X-ray energy range uncovered by the transfer function visualisation.

Contents

1 B07c Beamline Layout 1

2 Transfer Functions 1

3 600 l/mm Grating Ray-Tracing Results 4

4 B07c Grating Properties 7

5 Energy Range Optimisation Algorithm 7

List of Figures

1 Schematic of the B07c beamline. Distances are given from/to the centre of the optic in question. Acronyms used: VFM, vertically focussing mirror. HFM, horizontally focussing mirror. Figure reproduced from [1]. . . . 1

2 The transfer function plot for B07c PGM with the Pt mirror and 400 l/mm grating at c_{ff} = 1.05. Note the increasing behaviour of the transfer function for Fictitious Slits 1 (purple) between the dashed lines at energies 300 and 1120 eV. . . . 1

3 The B07c PGM at a c_{ff} of 1.05 at three different energies: 300, 700, and 1200 eV. Complete blocking is observed at 300 eV, partial blocking at 700 eV, and no blocking at 1200 eV. . . . 2

4 The proposed transfer function plot for B07c PGM with the Pt mirror and 400 l/mm grating at c_{ff} = 1.4. Note no blockage occurs and slits transmit 100% of the rays. The fall-off of the grating transfer function, starting at ca. 1000 eV, is due to the over-illumination of the grating Fig. 5. The dotted red vertical line is at 320 eV, which corresponds to the Pt *N*-edge and Rh *M*-edge absorptions. . . . 3

5 B07c PGM side-view at c_{ff} = 1.4, 1000 eV, where the beam footprint is becoming too large for the grating. The beam height drawn is 6.38 mm, as calculated by *SHADOW3* for this energy. . . . 4

6 **(a), (b)** Simulated Detector Flux for the 600 l/mm grating at c_{ff}= 1.4 with the measured flux. Both plots show the same data except where a is on a linear scale and b logarithmic scale. **(c), (d)** Simulated Detector Flux for the 600 l/mm grating at c_{ff}= 2.0 with the measured flux. Both plots show the same data except that a is on a linear scale and b logarithmic scale. . . . 5

7 **(a):** plots the simulated total flux (red) as a function of energy, along with the simulated first order (purple dashed) and the measured flux (green crosses). **(b):** the ratio of the simulated flux to the measured flux as a function of energy. . . . 6

8 The beamline energy resolutions obtained using the optimisation algorithm for a PGM with c_{ff} = 1.4, using the 400 l/mm grating in first order with an exit slit size 800 by 100 microns horizontally and vertically respectively. . . . 8

List of Tables

1 The properties of the B07c gratings used in our simulations. The clear aperture describes the size of the grating surface, with the tangential dimension being the dimension parallel to the rays and the sagittal perpendicular. c/d is the ratio of the height of the laminar groove c to the distance between each groove d. 7

1 B07c Beamline Layout

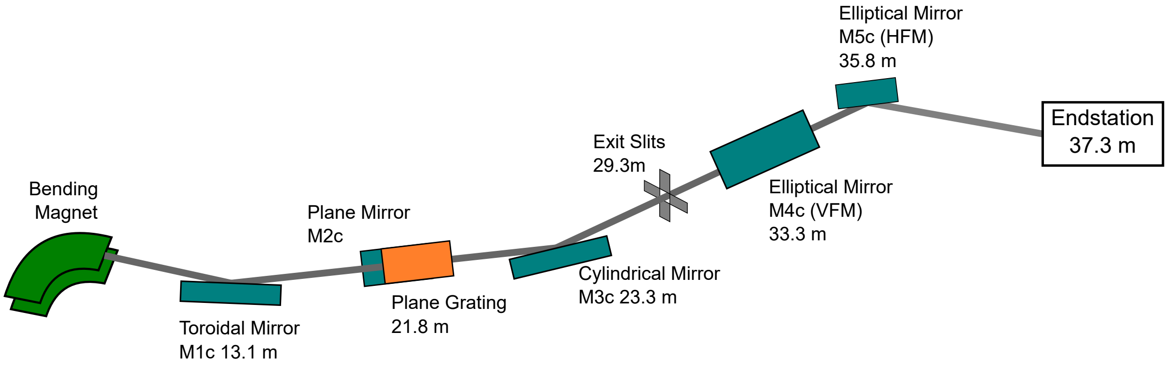

Figure 1: Schematic of the B07c beamline. Distances are given from/to the centre of the optic in question. Acronyms used: VFM, vertically focussing mirror. HFM, horizontally focussing mirror. Figure reproduced from [1].

2 Transfer Functions

As with any newly developed methodology, it is necessary to confirm if it produces physically sensible results. Results are presented here for the platinum-coated PGM mirror and the 400 l/mm grating combination. First, to verify that blocking has been implemented correctly, a *transfer function* as a diagnosis tool is proposed.

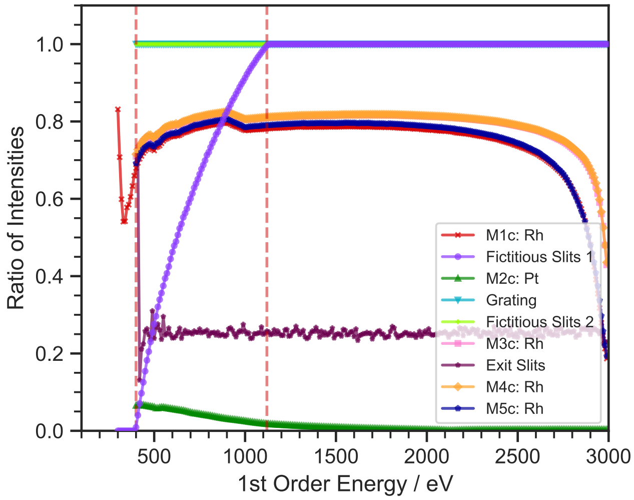

Figure 2: The transfer function plot for B07c PGM with the Pt mirror and 400 l/mm grating at c_{ff} = 1.05. Note the increasing behaviour of the transfer function for Fictitious Slits 1 (purple) between the dashed lines at energies 300 and 1120 eV.

For validation, the number of rays as well as the total intensity after each OE is stored. The intensity

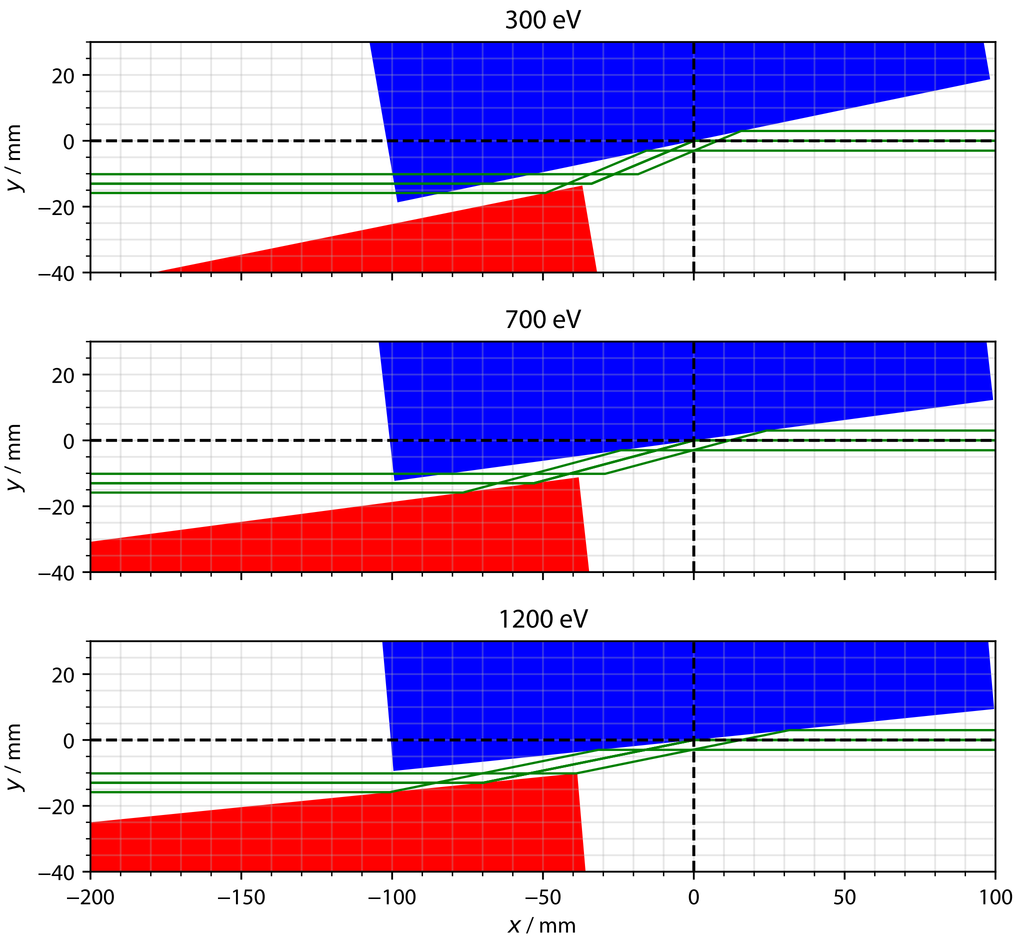

Figure 3: The B07c PGM at a c_{ff} of 1.05 at three different energies: 300, 700, and 1200 eV. Complete blocking is observed at 300 eV, partial blocking at 700 eV, and no blocking at 1200 eV.

being the magnitude of the electric (**E**) and magnetic (**B**) fields summed in quadrature ($\sqrt{|\mathbf{E}|^2 + |\mathbf{B}|^2}$). The transfer function plots the relative ratios of the intensities between two adjacent OEs, *i.e.* M1c/Bending Magnet, Fictitious slit 1/M1c... as a function of energy. This isolates the effect of the OE in question and allows the visualisation of its effect and its effect alone, highlighting isolated effects that are energy dependent, , *i.e.* geometrical and reflectivity related. An example of this is observed for M1c, a rhodium mirror that exhibits lower reflectivity above the rhodium *L*-edge (E $>$ 2800 eV). For the B07c PGM, if operated at c_{ff} = 1.05, a blockage by the grating will occur. The transfer function is presented in Fig. 2 with side view diagrams shown in Fig. 3 produced using PGMweb [2].

From Fig. 3, we can anticipate that the transfer function for Fictitious Slit 1 should show that the relative intensity at the first slits is zero below ca. 300 eV and increases until unity at ca. 1200 eV. This is clearly observed in Fig. 2. An intensity drop is observed for M4c, M3c, and M1c, which are all rhodium

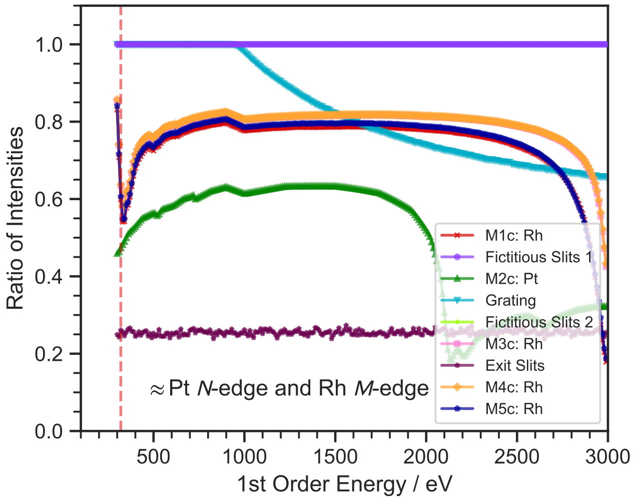

Figure 4: The proposed transfer function plot for B07c PGM with the Pt mirror and 400 l/mm grating at c_{ff} = 1.4. Note no blockage occurs and slits transmit 100% of the rays. The fall-off of the grating transfer function, starting at ca. 1000 eV, is due to the over-illumination of the grating Fig. 5. The dotted red vertical line is at 320 eV, which corresponds to the Pt *N*-edge and Rh *M*-edge absorptions.

mirrors, at approximately 3000 eV, which corresponds to the rhodium *L*-III edge at 3004 eV [3]. Due to the large angle of incidence at this c_{ff}, the efficiency of the platinum mirror has deteriorated so much that the absorption edge cannot be identified in this graph. Typically, high reflectivity from mirrors at normal incidence can only be realistically achieved for light in the visible and ultraviolet regions. In soft X-ray applications, the angle of incidence (relative to the normal) must be as close to 90° as possible to maintain maximum reflectivity [4]. Due to the energy optimisation step previously introduced, the exit slit ratio remains constant; the stochasticity comes from the optimisation algorithm, which can also be observed in the scatter in Fig. 8 in §5.

However, at a more typical c_{ff} of 1.4, which the beamline operates at, the platinum *M*-edge (2122 eV [3]) absorption edge can be clearly identified in Fig. 4. The gold coating of the grating has absorption edges that overlap closely with those of platinum, as they are adjacent to each other on the periodic table.

The fall-off of the grating transfer function is observed to start at just below 1000 eV (blue line in Fig. 4). This can be explained by the fact that the grating is over-illuminated, causing the flux to deteriorate, as shown in Fig. 5. As the energy increases above 1000 eV at c_{ff} = 1.4, the grating incidence angle will become smaller and smaller, increasing the over-illumination.

We note that for consistency, one would ideally scale the transfer function for the grating found using *SHADOW3* with the grating efficiency (computed separately using *MLgrating* [5]), but in practice

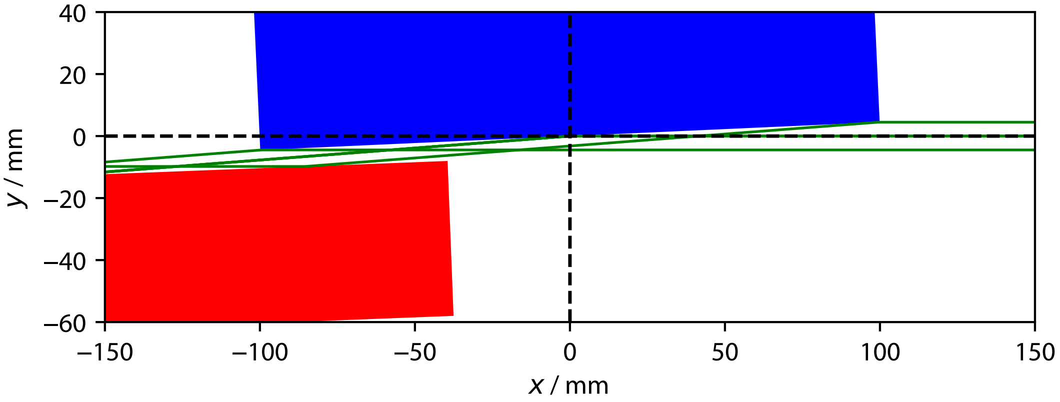

Figure 5: B07c PGM side-view at c_{ff} = 1.4, 1000 eV, where the beam footprint is becoming too large for the grating. The beam height drawn is 6.38 mm, as calculated by *SHADOW3* for this energy.

we have found that this substantially worsens the legibility of the transfer function plots, as the grating efficiency often has a complicated dependence of the photon energy and can therefore obscure any geometrical effects.

In performing a fine-energy scan in the soft X-ray range, we have also uncovered a potential issue within the xraylib library used by *SHADOW3* to compute mirror reflectivities. That is, the linear interpolation used by Elam *et al.* to connect two data sets of scattering factors produces unphysical results at around 1000 eV. This highlights the need for a more robust X-ray optical constant database for the soft X-ray region, with the Henke tables [6] being a good candidate.

3 600 l/mm Grating Ray-Tracing Results

In the configuration with the 600 l/mm grating for B07c, the higher-order contamination is discernibly less prominent compared to the 400 l/mm grating, Fig. 6. Again, the second-order contribution is mainly in the lower energy range of 500-1100 eV. The total flux is generally comparable, but for a c_{ff} value of 2.0, the first-order flux for the 400 l/mm grating is less than half of the total weighted flux, compared to 600 l/mm where the first order is ca. 70% of the total flux at 1000 eV. In addition, the peak flux of the 600 l/mm grating is at ca. 1500 eV and is roughly twice that offered by the 400 l/mm grating at 800 eV. The obvious recommendation would be for the beamline to use the 600 l/mm grating in all instances where the desired energy is above ca. 500 eV.

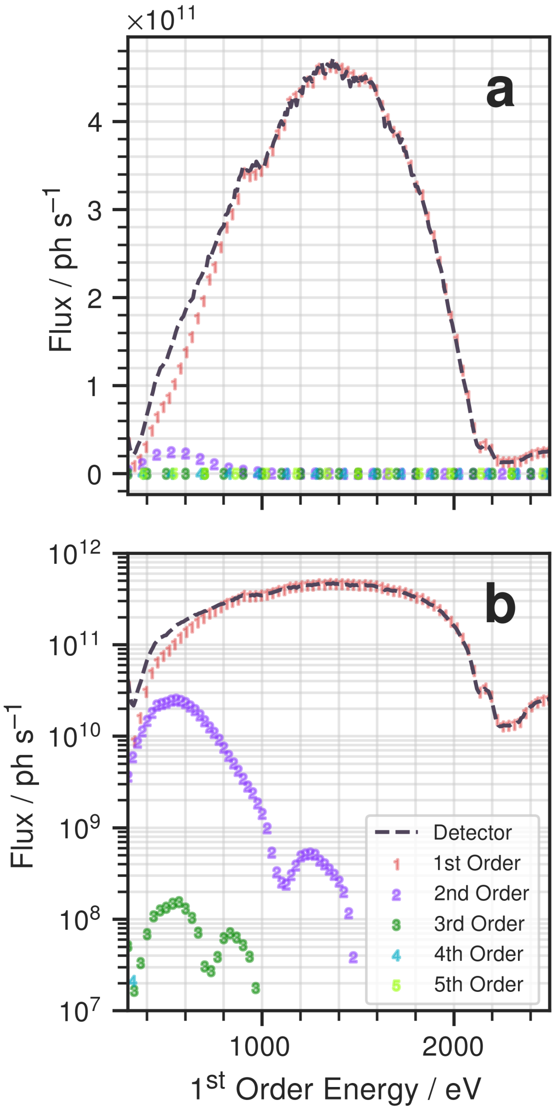

Figure 6: **(a), (b)** Simulated Detector Flux for the 600 l/mm grating at c_{ff}= 1.4 with the measured flux. Both plots show the same data except where a is on a linear scale and b logarithmic scale. **(c), (d)** Simulated Detector Flux for the 600 l/mm grating at c_{ff}= 2.0 with the measured flux. Both plots show the same data except that a is on a linear scale and b logarithmic scale.

The 600 l/mm grating simulation results show very similar behaviour to the measurements [1] as shown in Fig. 7.

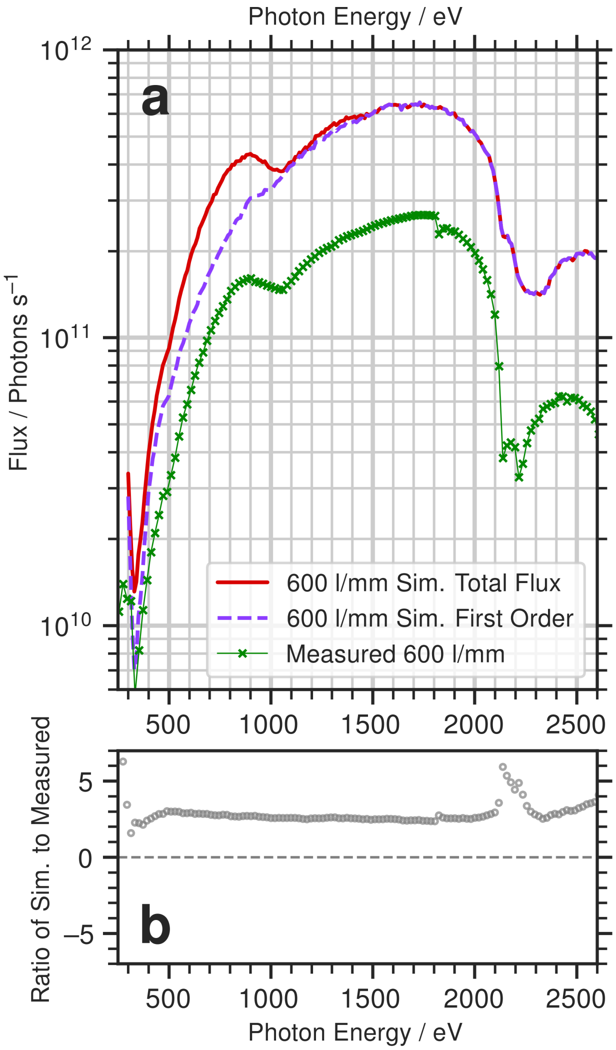

Figure 7: **(a):** plots the simulated total flux (red) as a function of energy, along with the simulated first order (purple dashed) and the measured flux (green crosses). **(b):** the ratio of the simulated flux to the measured flux as a function of energy.

4 B07c Grating Properties

Table 1: The properties of the B07c gratings used in our simulations. The clear aperture describes the size of the grating surface, with the tangential dimension being the dimension parallel to the rays and the sagittal perpendicular. c/d is the ratio of the height of the laminar groove c to the distance between each groove d.

Property	400 l/mm Grating (Laminar)	Property	600 l/mm Grating (Blazed)
Clear Aperture (tangential $\times$ sagittal)	190 mm $\times$ 17 mm	Clear Aperture (tangential $\times$ sagittal)	190 mm $\times$ 30 mm
Coating Material	40 nm of Au	Coating Material	30 nm of Au
Substrate Material	Si	Substrate Material	Si
Line Density	400 lines /mm	Line Density	600 lines / mm
Groove height	11.8 nm	Blaze Angle	0.49°
c/d ratio	0.676	Anti-blaze Angle	175.62°
Aspect angle	7.6°	–	–

5 Energy Range Optimisation Algorithm

If a PGM is configured with a geometry to transmit a certain energy of X-rays at a particular c_{ff}, the main energy range of interest is significantly smaller compared to the whole operating range of the photon source. Rays of energies not an integer multiple of 400 eV are not transmitted by the PGM and are therefore wasted. Due to the stochastic nature of SHADOW, rays are generated across the source energy. This makes calculation inefficient, as only rays within a small energy range are transmitted. However, the selected energy range of the source should also not be too small, where it would become the limiting factor, and thus, the energy resolution of the beamline cannot be derived. We present some of the beamline energy resolutions found in this work in Fig. 8. As explained in the main text, the energy range of the source for each incident energy was set to 2ΔE.

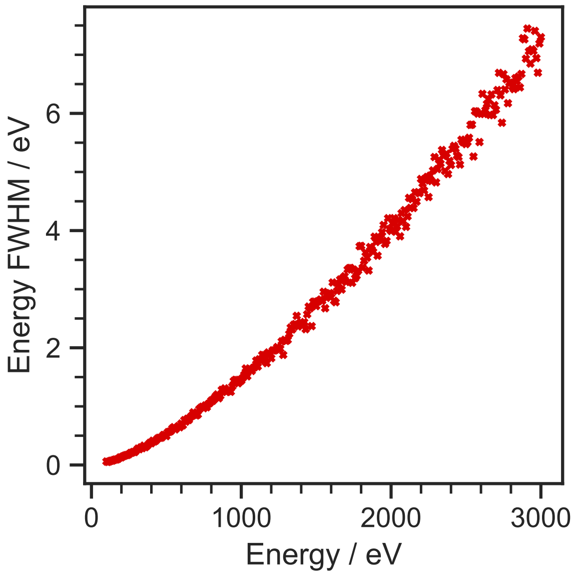

Figure 8: The beamline energy resolutions obtained using the optimisation algorithm for a PGM with c_{ff} = 1.4, using the 400 l/mm grating in first order with an exit slit size 800 by 100 microns horizontally and vertically respectively.

References

[1]G. Held, F. Venturini, D. C. Grinter, P. Ferrer, R. Arrigo, L. Deacon, W. Quevedo Garzon, K. Roy, A. Large, C. Stephens, A. Watts, P. Larkin, M. Hand, H. Wang, L. Pratt, J. J. Mudd, T. Richardson, S. Patel, M. Hillman, and S. Scott, "Ambient-pressure endstation of the versatile soft x-ray (versox) beamline at diamond light source", J. Synchrotron Radiat. **27**, 11531166 (2020).

[2]P. Y. Wang, M. Bazan da Silva, M. Hand, H. Wang, P. Chang, V. Beilsten-Edmands, T. K. Kim, T.-L. Lee, K. Sawhney, and A. C. Walters, "*PGMweb*: an online tool for visualizing the X-ray beam path through plane grating monochromators", J. Synchrotron Radiat. **32**, 261–268 (2025).

[3]W. M. Haynes and D. R. Lide, eds., *Crc handbook of chemistry and physics, A ready-reference book of chemical and physical data*, 96. ed., 2015-2016, Includes bibliographical references and index (CRC Press, Boca Raton, Fla. [u.a.], 2015).

[4]W. B. Peatman, *Gratings, mirrors and slits, Beamline design for soft x-ray synchrotron radiation sources*, Description based on publisher supplied metadata and other sources. (CRC Press LLC, Boca Raton, 1997), 1227 pp.

[5]A. Walters, S. Wen, Q. Huang, Z. Wang, H. Wang, and K. Sawhney, "MLgrating: a program for simulating multilayer gratings for tender X-ray applications", J. Synchrotron Radiat. **31**, 10.1107/s1600577524006271 (2024).

[6]B. L. Henke, E. M. Gullikson, and J. C. Davis, "X-ray interactions: photoabsorption, scattering, transmission, and reflection at e = 50-30,000 ev, z = 1-92", At. Data. Nucl. Data Tables **54**, 181342 (1993).
